# Newly Identified *Mycobacterium africanum* Lineage 10, Central Africa

**DOI:** 10.3201/eid3003.231466

**Published:** 2024-03

**Authors:** Christophe Guyeux, Gaetan Senelle, Adrien Le Meur, Philip Supply, Cyril Gaudin, Jody E. Phelan, Taane G Clark, Leen Rigouts, Bouke de Jong, Christophe Sola, Guislaine Refrégier

**Affiliations:** University Bourgogne Franche-Comté (UBFC), Besançon, France (C. Guyeux, G. Senelle);; Université Paris-Saclay–AgroParisTech, Gif-sur-Yvette, France (A. Le Meur, G. Refrégier);; Institut Pasteur de Lille Center for Infection and Immunity of Lille, Lille, France (P. Supply, C. Gaudin);; London School of Hygiene and Tropical Medicine, London, UK (J.E. Phelan, T.G. Clark, L. Rigouts, B. de Jong);; Université Paris-Saclay, Saint-Aubin, France (C. Sola);; Université Paris Cité, Paris (C. Sola)

**Keywords:** Mycobacterium tuberculosis, tuberculosis and other mycobacteria, TB, respiratory infections, bacteria, pathogen diversification, tuberculosis emergence, lineage 10, L10, central Africa, Democratic Republic of the Congo

## Abstract

Analysis of genome sequencing data from >100,000 genomes of *Mycobacterium tuberculosis* complex using TB-Annotator software revealed a previously unknown lineage, proposed name L10, in central Africa. Phylogenetic reconstruction suggests L10 could represent a missing link in the evolutionary and geographic migration histories of *M. africanum*.

The traditional view of restricted diversity among bacterial agents causing human and animal tuberculosis is being revised thanks to wide use of whole-genome sequencing (WGS). Besides *Mycobacterium canettii*, representative of exceptional, nonclonal, early-evolution branching lineages of tubercle bacilli in eastern Africa, several previously unknown lineages of *M. tuberculosis* complex have been identified in Africa during the past decade. *M. tuberculosis *complex lineage 7 (L7) was discovered in the Horn of Africa and L8 in the African Great Lakes region (*1*,*2*). *M. africanum *L9 was found only in Djibouti and Somalia. In contrast, 2 other major *M. africanum*–affiliated lineages contributing substantially to the tuberculosis burden, L5 and L6, are found mostly in western Africa (*3*). The pathway between eastern and western Africa in the evolutionary history of the bacillus remains unclear. We describe a newly identified sister lineage of L6 and L9 associated with central Africa and discuss implications for determining the evolutionary history of related *M. africanum *lineages L5, L6, and L9. We based research on publicly available data and thus required no ethics approval. 

## The Study 

We used the TB-Annotator platform (G. Senelle, unpub. data, https://www.biorxiv.org/content/10.1101/2023.06.12.526393v1) to integrate WGS data from 102,001 *M. tuberculosis* complex isolates in the National Center for Biotechnology Information (NCBI) public domain. This platform identifies genetic variations, including single-nucleotide polymorphisms (SNPs), regions of difference (RDs), and IS*6110* insertions, differentiating selected genomes from *M. tuberculosis* H37Rv. The TB-Annotator database also contains information on genotypic drug resistance and geographic location of variant isolation. 

SNPs from an exploratory set comprising 15,699 isolates largely of Africa origin were used to build a phylogenetic tree. Our analysis identified a lineage sister to *M. africanum* L6 and L9, branching between these lineages and the animal lineage A1 (La_A1) (*3*). The newly identified lineage is represented by only 2 genomes: ERR2707158, obtained from a strain isolated in 2008 from a patient residing in Kinshasa, Democratic Republic of the Congo (DRC), now incorporated under reference ITM-501386 (CT2008–03226) in the coordinated collections of microorganisms of the Institute of Tropical Medicine (Antwerp, Belgium); and ERR2516384, obtained from a strain isolated in Belgium in 2013 (V. Mathys, pers. comm., email, 2023 Jul 5). The genomes of the new lineage carried none of the SNP markers described in the latest *M. tuberculosis *complex lineage classification scheme (4) and no SNPs that confer drug resistance. 

To confirm the phylogenetic position of those 2 genomes, we identified SNPs from 132 isolates covering the genetic and geographic diversity of L5 and L6 and including representatives of all other lineages using the Genotube pipeline (A. Le Meur, pers. comm., email, 2023 Sep 15) and TB-Profiler (*5*). Resulting phylogenetic reconstruction confirmed the clustering of ERR2707158 and ERR2516384 in a branch between L6 and L9 and animal lineage La_A1 (Figure). The newly designated L10 samples shared 375 specific SNPs with isolates from our selected set of 132 samples; 243/375 specific SNPs were not detected in any of the 102,001 genomes included in TB-Annotator. Among those specific SNPs, 91 were synonymous (Appendix 1). The pairwise distance between the 2 samples of interest was 382 SNPs (SNPs outside of repetitive regions, manually checked when discordant between 2 pipelines), much shorter than the distance to the other samples of our selection (minimum 1,137 SNPs; average 1,591 ±222 SNPs) (Appendix 2, Figure 1). 

**Figure:**
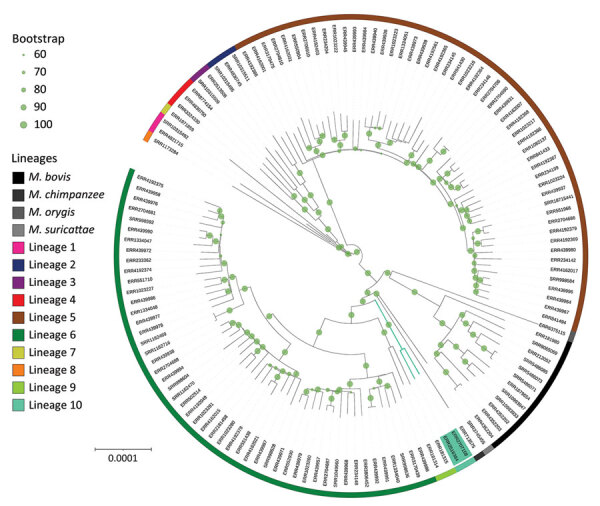
Global *Mycobacterium *phylogeny including newly identified *M. africanum* L10 (proposed) strains (green shading). We selected *M. africanum* samples for harboring RD9 deletion, having documented country of origin (for the purpose of additional analyses; Appendix 2, Figure 2), and refined our selection to retain a sole representative of each sublineage for each country. This sample represents representing the genetic and geographic diversity of *M. africanum* in Africa. Specifically for this phylogenetic reconstruction, single-nucleotide polymorphisms were identified in comparison with an *M. tuberculosis* ancestor (*11*) and reincorporated into the whole genome to avoid biases in the molecular model or need for Lewis correction. Phylogeny was rooted with *M. canettii,* subsequently removed for better visualization. Bootstrap support was computed using 100 replicates and shown when ≥0.6. Circles confirm the large support of almost all branches, especially of L10 and its sister branches. L10 branching point lies between L9 and the La_A1 lineage grouping chimpanzee and Dassie bacillus. Scale bar indicates nucleotide substitutions per site.

We next explored other features of the genomes to corroborate SNP-based phylogenetic inferences. In addition to the deletion of RD9 shared with the L5/L6 branch and animal-associated lineages, the 2 L10 genomes lacked RD7, RD8, and RD10 (*3*). However, they did not show the RD702 (L6/L9) or RD713 (L5) deletions. In contrast, the 2 unclassified genomes harbored the same specific large 9,134 nt deletion (*Rv0613c*–*Rv0622*) in *M. tuberculosis* H37Rv (NC\_000962.3:706602–715736) not observed in any other lineage. This segment included the toxin/antitoxin gene pair *vapB29*/*vapC29*. Two other shared deletions encompassed *eis* and *dnaE2* (Appendix 1), potentially limiting the ability to acquire aminoglycoside resistance (*6*) and possibly affecting some mutational properties (*7*) of those *M. africanum* strains. The 2 genomes also shared 4 IS*6110* copies at a position found in no other lineage (Appendix 1). In the CRISPR locus of the 2 L10 genomes, reconstructed using CRISPRbuilder-TB (*8*), we found the same absence of spacers 7 and 9 (43-spacer spoligotype format) seen in L6, L9, and La_A1 (Table) and all last spacers starting from spacers 22 (ERR2516384) or 26 (ERR2707158) (Table). 

**Table:** Spoligotype patterns of newly identified *Mycobacterium africanum* L10 (proposed) strains from central Africa compared with representative strains of L6, L9, and A1 lineage*

ID	Source†	No.	Spoligotype binary	SIT	Country of isolation
L10-BEL04200301729	SITVITWEB	1	■■■■■■□■□■■■■■■■■■■■■□□□□□□□□□□□□□□□□□□□□□□	Orph	Republic of Congo
L10-ERR2516384	TB-Annotator	1	■■■■■■□■□■■■■■■■■■■■■□□□□□□□□□□□□□□□□□□□□□□	Orph	Belgium‡
L10-ERR2707158	TB-Annotator	1	■■■■■■□■□■■■■■■■■■■■■■■■■□□□□□□□□□□□□□□□□□□	Orph	DRC
L6_SIT181	SITVITWEB	208	■■■■■■□□□■■■■■■■■■■■■■■■■■■■■■■■■■■■■■□■■■■	181	Gambia
L9	Coscolla 2021	3	■■■■■■□■□■■■□□□□□□□□□□□□□□□□□□□□□□■■■■□■■■■	Orph	Somalia
L9	Coscolla 2021	1	■■■■■■□■□□□□□□□□□□□□□□□□■■■■■■■■■■□■■■□■■■■	U	Djibouti
L9_FXX01199901706	SITVITWEB	1	■■■■■■□■□□□□□□□□□□□□□□□□■■■■■■■■■■□■■■□■■■■	Orph	France
NLD009501731	SITVITWEB	2	■■■■■■□■□■■■□□□□□□□□□□□□□□□□□□□□□□■■■■□■■■■	Orph	Netherlands
A1_Dassie bacillus	TB-Annotator	1	■■■■■■□■□■■■□□■■■■■□□□□□□□□□□□□□□□□■■■□□■■■	U	South Africa
A1_*M. mungi*	https://mbovis.org, TB-Annotator	1	■■□■■■□■□■■□□□□□□□□□□□□□□□□□□□□□□□□□■■□■■■■	SB 1960	Botswana
A1_chimpanzee bacillus	Coscolla 2013, TB-Annotator	1	■■■□□□□□□□□□□□□□□□□□□□□□□□□□□□□■■■■■■■□■□□■	U	Côte d’Ivoire

*L6, L9, and both L10 samples harbor the same losses of spacer 7 and of spacer 9. DRC, Democratic Republic of the Congo; orph, orphan (single reported occurrence); SIT, spoligotype international type; U, undesignated. †Coscolla 2021, (*3*); Coscolla 2013, (*12*); SITVITWEB, (*9*); TB-Annotator, G. Senelle, unpub. data, https://www.biorxiv.org/content/10.1101/2023.06.12.526393v1. ‡Origin unknown.

The genetic features of the strains we identified, combining outlying phylogenetic position, genetic distance from the L6/L9 branch and other known *M. tuberculosis* lineages, distinctive regions of deletions and IS*6110* insertions, and specific spoligotype signatures, led us to propose their classification in a newly designated L10 lineage. We propose 3 synonymous SNPs (*gyrA* G7901T, *recN* C1920096T, and *dnaG* C2621730T) compared with the H37Rv 000962.3 reference sequence in housekeeping genes to identify the new lineage. 

To evaluate potential regional and global circulation of L10 strains, we searched for similar spoligotype patterns using SITVIT2, which accumulates spoligotypes from >110,000 isolates from 131 countries (*9*). We identified a single instance, BEL04200301729, showing the same spoligotype pattern as ERR2516384, which might represent a third occurrence of L10. Of note, that strain was isolated in the Republic of the Congo, a country neighboring DRC, where ERR2707158 was collected (Appendix 2, Figure 2). We also browsed spoligotyping results from next generation sequencing data, collected from ≈1,500 isolates from a 2016–2017 national survey in DRC, targeted using Deeplex Myc-TB (https://www.deeplex.com) (*10*) but detected no similar pattern. Thus, both global (TB-Annotator and SITVIT2) and local (*10*) datasets suggested that L10 strains are rare at the worldwide level, and aside from migratory dissemination, likely restricted to central Africa. Mapping of *M. africanum* diversity in Africa showed that in addition to L10, central Africa also hosts a relatively large diversity of L5 strains (Appendix 2, Figure 2). 

Despite the rarity of L10, its specific phylogenetic positioning and presence in central Africa provide new elements to the complex evolutionary history of *M. africanum*. Currently, the most likely scenario favors western Africa as the place of origin of all *M. africanum* variants (*3*). This scenario implies that L5 and L6 ancestors emigrated from eastern Africa and diversified in western Africa and that L9 migrated back to eastern Africa. Finding L10 in central Africa with intermediate branching between L5 and L6/L9 can fit this scenario but adds an independent migration from western Africa to central Africa. Alternatively, *M. africanum* could have emerged close to central Africa and subsequently migrated westwards and eastwards. This alternative scenario, however, would require greater sampling in central regions of Africa to gain real support. 

## Conclusions

Through the extensive mining of WGS and genotyping databases, we newly identified a thus far rare *M. tuberculosis* complex lineage, L10 (proposed), present in central Africa. The lineage is characterized by a new region of deletion, IS*6110* insertions, and 243 SNPs, including *gyrA* G7901T, *recN* C1920096T, and *dnaG* C2621730T. L10 represents a sister clade to L6, found mainly in western Africa, and L9, specifically in eastern Africa, and reveals a putative previously missing piece in the evolutionary history and migrations of *M. africanum*. Our findings extend the known diversity of *M. africanum *in Africa. 

Appendix 1 Additional information about genomic details for newly identified *M. africanum *lineage L10 in Central Africa.

Appendix 2 Additional information about phylogenic relatedness and geographic circulation of newly identified *M. africanum *lineage L10.
