# Supplementary material for: Newly Identified Mycobacterium africanum Lineage 10, Central Africa
Source: Emerg Infect Dis. 2024 Mar;30(3):560–3. doi: 10.3201/eid3003.231466 (PMC10902520; doi:10.3201/eid3003.231466)
Supplement: Appendix 2 — Additional information about phylogenic relatedness and geographic circulation of newly identified M. africanum lineage L10. [file 23-1466-Techapp-s2.pdf]

Article DOI: https://doi.org/10.3201/eid3003.231466

EID cannot ensure accessibility for Appendix materials supplied by authors. Readers who have difficulty accessing Appendix content should contact the authors for assistance.

Newly Identified *Mycobacterium africanum* Lineage 10, Central Africa

Appendix 2

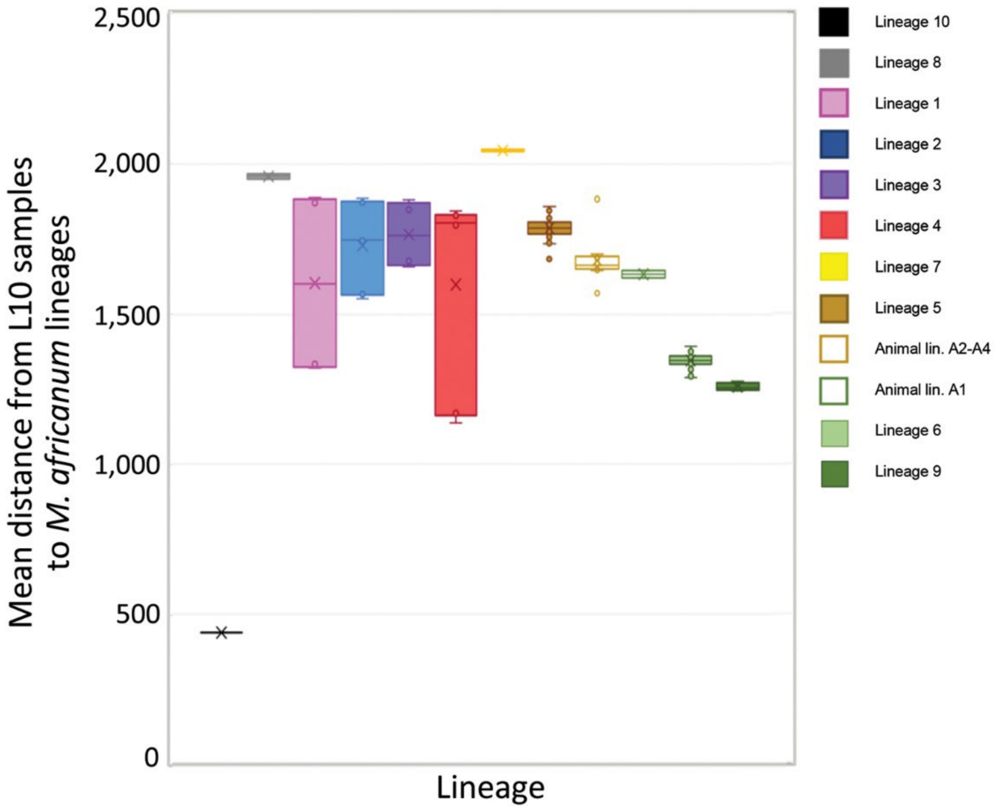

Appendix 2 Figure 1. Intra- versus inter-lineage genetic distance of L10 strains. The distances between L10 strains and all other samples shown in the tree of Figure 1 were computed using snp-dist tool excluding PE-PPE regions. The legend is provided in the same order as they appear on the X-axis. Note that L10 strains are much more similar to one another than to strains from other lineages.

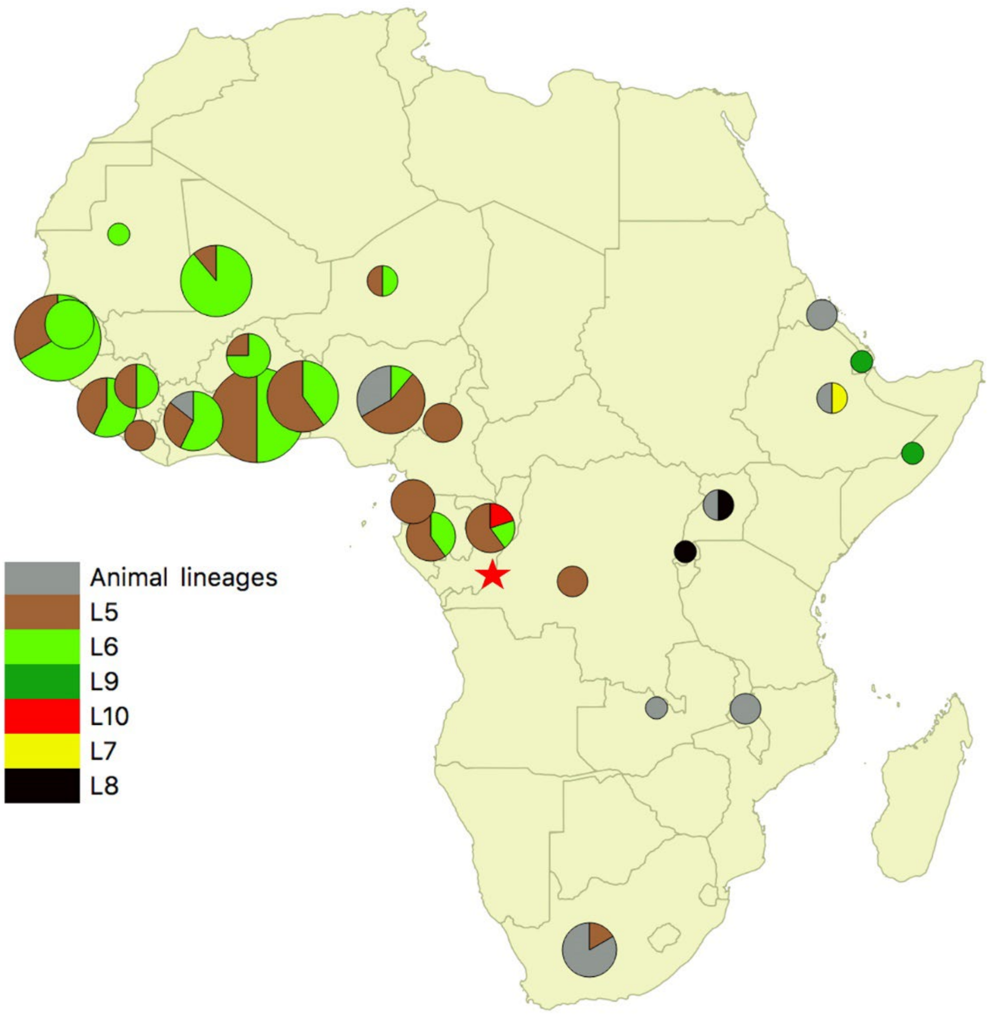

Appendix 2 Figure 2. Map of Africa showing distribution of *Mycobacterium africanum* sublineages. This map locates the African samples from our 132-sample diversity set (Figure 1 in the main text), as annotated by Genotube. Animal lineages include La_A1 and other animal lineages. The sampling of these lineages should not be considered representative because of lack of sublineage definition among animal lineages in Genotube. In contrast, because 1 sample from each known *M. africanum* sublineage was retained for each country, sampling can be considered as representative of L5 and L6 diversity. The map was built with QGIS version 3.4.6 (Madeira on Mac-OS10.13.6, using an open source shapefile with African boundaries obtained from https://hub.arcgis.com). The single localized L10 strain (from Democratic Republic of Congo) reported in the NCBI SRA database is shown in red. The red asterisk shows the Republic of Congo where another L10 strain was isolated according to SITVITWEB database. Of note, Belgium, where the third L10 strain was collected, has a history of colonizing DRC; the strain was isolated from a patient with an African name which is not indicative but compatible with the actual origin of this strain in central Africa (DRC). This map also highlights the presence of L5 in central Africa.
